# The Impact of Language Barriers on Dental Anxiety in Pediatric Patients: A Feasibility Pilot Study

**DOI:** 10.3390/dj14070448

**Published:** 2026-07-17

**Authors:** Avia Fux-Noy, Yonatan Nahum, Aviv Shmueli, Elinor Halperson

**Affiliations:** 1 Department of Pediatric Dentistry, Faculty of Dental Medicine, Hebrew University of Jerusalem, Jerusalem 9112102, Israel; aviv.shmueli@mail.huji.ac.il (A.S.); elinor.halperson@mail.huji.ac.il (E.H.); 2 Department of Pediatric Dentistry, Dental Medicine, Hadassah Medical Center, Jerusalem 9112102, Israel; yonatan.nahum@mail.huji.ac.il

**Keywords:** language barriers, dental anxiety, pediatric patients, translation, interpretation

## Abstract

**Background/Objectives**: Effective communication between practitioners and their patients is pivotal to achieving successful treatment outcomes. The objective was to evaluate differences in post-examination anxiety and intra-operative behavior among pediatric patients when examined by a language-concordant dentist versus one with whom there was a language barrier. **Methods**: This controlled prospective pilot study included 60 children aged 4–10 years, divided into two groups: a control group consisting of patients and dentists who spoke the same language, and a study group comprising patients and dentists with discordant primary languages. Anxiety levels were measured using the Facial Image Scale before and after dental examination, and behavior was assessed by the clinician using the Frankl Behavioral Rating Scale. **Results**: The groups were similar in mean age, gender distribution, and baseline anxiety. No significant differences were found between the groups in post-examination anxiety levels (*p* = 0.99), behavior during clinical examination (*p* = 0.871), or behavior during radiographic examination (*p* = 0.615). However, within the study group, significant correlations were found between higher pre-examination anxiety and more negative behavior during clinical examination (*p* = 0.045), as well as between higher post-examination anxiety and more negative behavior during clinical examination (*p* = 0.029); these correlations were not observed in the control group. **Conclusions**: Language differences between the dentist and the patient did not increase anxiety or negative behavior in children during dental examinations within this sample. Non-verbal communication and the presence of a translating parent may be important mediating factors in reducing anxiety among pediatric patients when language barriers exist. Future larger, well-controlled studies are needed.

## 1. Introduction

Effective communication between practitioners and their patients is pivotal to achieving successful treatment outcomes [1,2]. In pediatric dentistry, this is not merely important but imperative; an unpleasant dental visit can have adverse, long-lasting effects on a child’s willingness to seek care and their oral health [3]. Dental anxiety is a common phenomenon affecting approximately 30% of children globally and represents a significant challenge in pediatric dental practice [4]. It is important to distinguish between dental anxiety, an emotional state characterized by apprehension about dental treatment, and dental behavior management problems, which refer to uncooperative or disruptive behaviors during dental procedures. While these concepts are related, they are not identical; anxious children do not always exhibit behavioral problems, and behavioral issues may arise from factors other than anxiety [5].

Recognizing the developmental characteristics unique to pediatric patients is essential, particularly regarding their specific communication needs. Communication in the pediatric setting requires fostering sustained trust, addressing the patient’s challenges, and facilitating clinical procedures by tailoring linguistic approaches to the child’s developmental stage [6]. Effective communication comprises two essential components: verbal and non-verbal. The pediatric dentist utilizes these tools to manage the child’s emotions and behavior toward a positive and cooperative state [7,8]. Evidence-based nonpharmacological behavior guidance techniques, including modeling, positive reinforcement, breathing relaxation, and cognitive behavioral therapy, have demonstrated substantial effects in reducing dental anxiety in children [8,9,10]. However, when a language barrier impedes verbal communication, the dentist may encounter difficulty in establishing a trusting relationship with the child and implementing these behavior guidance techniques effectively.

In multilingual societies, it is common for pediatric dentists to encounter language barriers when treating young patients. According to Eurostat data, almost 10% of people living in European Union (EU) countries were non-nationals, with 3.1% being citizens of another EU country, and 6.4% being citizens of a non-EU country [11]. Research conducted in US dental school clinics found that nearly a quarter of patients had limited English proficiency [12]. Compared to English-speaking patients, those with limited English proficiency experience significant disparities in medical and oral health outcomes, including reduced access to care and lower service utilization [13]. These patients report lower satisfaction with medical encounters and receive fewer clinical explanations and follow-up instructions [14].

Dentists may attempt to bridge the communication gap by learning key words or by relying on third-party interpreters, such as professional translators, dental assistants, or bilingual parents. However, the use of untrained interpreters presents significant challenges, as they are more likely to make errors, violate confidentiality, and increase the risk of poor outcomes [15]. Untrained interpreters may fail to provide precise translations or may offer their own interpretations. This is particularly problematic in pediatric dentistry, where the use of euphemisms, gentle substitutes for potentially frightening words, is considered one of the most common behavior guidance techniques for young patients [16]. When an unprofessional interpreter, such as a parent, translates for the dentist, they often mistranslate these euphemisms or use terms pediatric dentists deliberately avoid, which can inadvertently trigger anxiety or fear. Furthermore, parental dental anxiety is well-documented to strongly correlate with, and directly exacerbate, children’s anxiety during clinical encounters [17,18,19]. Consequently, an anxious parent acting as an interpreter may unintentionally transmit their own apprehension to the child.

To date, there is a gap in the literature regarding pediatric behavioral responses to dental care in the context of language barriers between the dentist and the patient. Thus, the primary objective of this study was to evaluate differences in post-examination anxiety and intra-operative behavior among pediatric patients when treated by a language-concordant dentist versus one with whom a language barrier exists. The secondary objectives were: (1) to examine the impact of an interpreter’s presence on a child’s anxiety and behavior; (2) to evaluate whether the interpreter’s identity (e.g., a parent versus another third-party interpreter) influences the child’s anxiety and behavior; and (3) to assess whether additional variables, such as age or gender, affect a child’s anxiety and behavior in the context of language disparities with the clinician. The hypothesis tested in this study is that when a pediatric patient does not speak the same language as the dentist, their anxiety levels will be higher and their behavior will be more negative compared to children who are language-concordant with their dentist.

## 2. Materials and Methods

### 2.1. Study Design and Participants

This controlled prospective feasibility pilot study was conducted at the Department of Pediatric Dentistry at Hadassah Medical Center, Jerusalem, Israel. Parents of children attending the department for dental examinations were invited to participate. Comprehensive information was provided in plain, non-technical language, and written informed consent was obtained from the parents or guardians of all participants. Providing care to children by clinicians who do not speak their primary language is a routine practice in the department and was not implemented specifically for this study. The assignment of patients to various clinicians was based on provider availability rather than study requirements. The study protocol was approved by the Institutional Human Subjects Ethics Committee of Hadassah Medical Center (HMO-0234-24). The recruitment timeframe spanned from 4 August 2024 to 21 April 2025.

The patient population attending the department’s clinic is diverse, representing a wide range of primary languages, including Hebrew, Arabic, English, Russian, Amharic, and Yiddish. Participants were categorized into two groups based on language concordance between the child and the clinician: a control group, consisting of patients and dentists speaking the same language, and a study group, comprising patients and dentists with discordant primary languages. Inclusion criteria were healthy children aged 4–10 years with a baseline dental anxiety score of 1–3 on the Facial Image Scale (FIS) [20]. Children under age 4 were excluded because they are in a developmental stage characterized as “pre-cooperative” or “lacking in cooperative ability” [21]. At this stage, children may not yet possess the cognitive capacity to fully understand clinical situations or cooperate accordingly, which would make it difficult to isolate the specific influence of language on their behavioral responses. Exclusion criteria included children with systemic medical conditions, developmental disabilities, or high baseline dental anxiety (FIS scores of 4–5). Children with high baseline anxiety were excluded to prevent extreme emotional states from confounding the study results.

### 2.2. Study Procedure

Initially, the primary investigator assessed each child’s baseline anxiety level using the FIS. Only children meeting the inclusion criteria for baseline anxiety participated in the study. The FIS consists of five illustrations of faces ranging from very happy to very worried; the child was asked to point to the face that best represented their current feelings. This scale has been validated for use in children as young as 3 years old [20]. The question was introduced in the child’s spoken language. Following the baseline assessment, the children underwent a clinical dental examination in a dental chair using standard overhead lighting, a dental mirror, and a probe in the presence of their parents. Radiographs were taken as clinically indicated. Patient behavior during the clinical and radiographic examination was evaluated by the treating dentist using the Frankl Behavioral Rating Scale [7]. The examinations were performed by several pediatric dentistry residents, all of whom were skilled and experienced in applying the Frankl scale. Upon completion of the examination, the primary investigator re-evaluated the child’s anxiety level using the FIS. Additional data were recorded, including patient age, gender, chief complaint, and whether it was the child’s first dental visit. Within the study group, the identity of the interpreter (dental assistant, parent, or no interpretation) was also documented.

### 2.3. Sample Size Calculation

A sample size calculation was performed using G*Power software (v3.1) for a two-tailed independent samples t-test. In the absence of prior specific literature, a conventionally large effect size (Cohen’s d = 0.80) was targeted to ensure clinical relevance. With an alpha level of 0.05 and 80% statistical power, a minimum of 52 participants (26 per group) was required.

### 2.4. Statistical Analysis

Data are presented as frequencies, means, and standard deviations (SD). Age was analyzed using an independent t-test, and gender distribution was analyzed using the Chi-square test. A comparative analysis of anxiety levels before and after the examination between the groups was performed using the Mann–Whitney U test. Spearman’s rank correlation coefficient was used to examine the relationship between behavior during the examination and anxiety levels both before and after the dental examination. Within the study group, the association between anxiety levels and the presence of an interpreter was assessed using the Mann–Whitney U test. All statistical calculations were performed using IBM SPSS Statistics software (version 28.0.). Statistical significance was set at *p* < 0.05. The complete raw dataset is available in the supplementary material (Table S1).

## 3. Results

**Figure 1:**
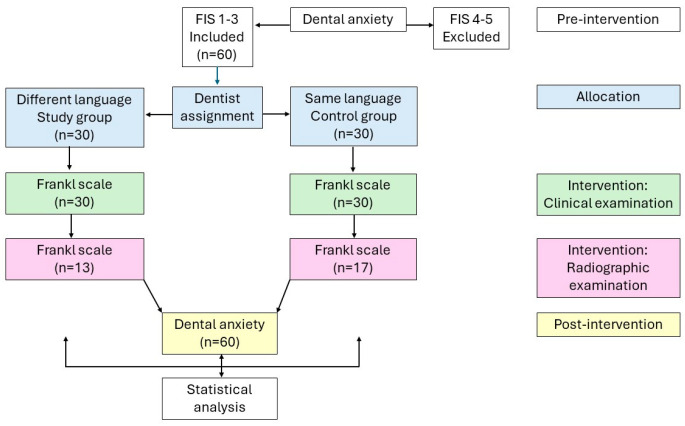
Study flowchart.

The study flowchart is presented in Figure 1. A total of 60 children participated in the study, with 30 assigned to the study group and 30 to the control group. Of the total sample, only one patient in the study group was attending their first dental visit; the remaining 59 children had prior dental experience. The examinations in both groups were conducted by ten dentists, seven of whom were fluent in Hebrew and English, while three were additionally fluent in Arabic. Within the study group, five children were Yiddish-speaking, one was Tigrinya-speaking, and the remaining participants were Arabic-speaking.

The study group consisted of 14 boys and 16 girls, with a mean age of 6.17 years (SD 1.91). The control group included 16 boys and 14 girls, with a mean age of 6.97 years (SD 1.92). No statistically significant differences were found between the groups regarding age (t = 1.616, *p* = 0.11) or gender (χ^2^ (1) = 0.267, *p* = 0.605) (Table 1). Regarding baseline anxiety, the study group had a mean score of 2.03 (SD 0.87), compared with 1.87 (SD 0.9) in the control group (U = 402.0, *p* = 0.478) (Table 1). These results indicate that the two groups were well-matched in terms of demographic and baseline variables, ensuring their comparability for subsequent analyses.

No statistically significant differences were observed between the groups regarding post-examination anxiety (U = 449.5, *p* = 0.99) or the degree of change in anxiety levels before and after the examination (U = 419.0, *p* = 0.646). Similarly, behavioral scores during the clinical examination did not differ significantly between the groups (U = 439, *p* = 0.871) (Table 1). Radiographic examinations were performed on 13 children in the study group and 17 children in the control group. Analysis of behavior during radiographic assessment likewise revealed no significant differences between the study and control groups (U = 98.5, *p* = 0.615) (Table 1).

Within the study group, a statistically significant negative correlation was observed between baseline anxiety and behavior (r_s_ = −0.368, *p* = 0.045), indicating that higher pre-treatment anxiety was associated with lower levels of cooperation. In contrast, no such correlation was found in the control group (r_s_ = 0.0034, *p* = 0.985). Furthermore, a significant negative correlation was identified in the study group between behavior and post-examination anxiety (r_s_ = −0.396, *p* = 0.029); however, this relationship did not reach statistical significance in the control group (r_s_ = −0.351, *p* = 0.056).

Within the study group, all interpreters were the children’s parents; therefore, it was not possible to evaluate the influence of the interpreter’s identity (e.g., parent vs. staff member) on anxiety and behavior. Furthermore, 27 children were examined in the presence of a bilingual parent who provided interpretation, while only three children were examined without any interpreter. Due to this highly unequal distribution and the extremely small sample size of the no-interpretation subgroup, a meaningful statistical comparison between parental interpretation and no interpretation could not be performed.

No statistically significant correlation between post-examination anxiety and age was found in either the control group (r_s_ = −0.091, *p* = 0.630) or the study group (r_s_ = −0.312, *p* = 0.093). No statistically significant differences in post-examination anxiety between boys and girls were observed in the control group (U = 110.0, *p* = 0.933) or the study group (U = 85.0, *p* = 0.261).

## 4. Discussion

The findings of the present study did not support the initial hypothesis. Within this sample, language barriers did not result in a significant increase in pediatric dental anxiety. Furthermore, no significant differences in clinical behavior were observed between children treated by a language-concordant dentist and those treated by a clinician with whom a language barrier existed. These results suggest that language discordance, in the context of a dental examination, may not be a primary determinant of anxiety or uncooperative behavior in children.

Communication between a clinician and a child is not limited to verbal language; it relies heavily on non-verbal cues, such as facial expressions, body language, tone of voice, eye contact, and physical touch [7,8]. These components may be paramount in mitigating anxiety and establishing a sense of security for the pediatric patient [22]. While non-verbal communication strategies were not formally documented in this study, it is plausible that these elements were sufficient to bridge the linguistic gap, which may account for the lack of significant differences in anxiety and behavior between the study and control groups.

Another potential explanation is the presence of an interpreter within the study group. The vast majority of children in this group received interpretation from a parent, with only three children undergoing the examination without any interpretation. It is noteworthy that ad hoc interpretation by a parent can, in some cases, inadvertently increase anxiety. Parents may lack the clinical expertise to interpret medical terminology or convey information in a manner that is developmentally appropriate for the child. This lack of proficiency can lead to confusion, misunderstandings, and missed opportunities to utilize anxiety-reduction techniques through tailored explanations. Studies demonstrate that the use of professional interpreters increases patient satisfaction, improves adherence and outcomes, and reduces adverse events [15]. However, family members and friends are commonly used as interpreters in healthcare settings, despite recommendations against this practice [23]. Moreover, the anxiety of the parent accompanying the child has been shown to directly affect children’s anxiety [19]. However, parental anxiety was not measured in the present study, which represents a potential confounding factor that could have influenced the behavioral outcomes. These findings highlight a need for future research to further investigate the differences between professional versus ad hoc interpreters, as well as the impact of no interpretation at all, on children’s anxiety and cooperation during dental treatment.

The study identified a significant correlation between behavior and both pre- and post-examination anxiety levels within the study group, whereas no such association was found in the control group. These findings suggest that in the presence of a language barrier, anxiety is more likely to manifest as negative behavior. Conversely, when the clinician and patient are language-concordant, anxiety does not necessarily result in uncooperative behavior. This distinction may be attributed to the fact that an anxious patient’s inability to understand the clinician further diminishes their capacity to cooperate, which in turn reinforces their anxiety by the end of the procedure. In the control group, however, even anxious children can understand the clinician’s instructions and reassurances, which likely facilitates better cooperation despite their distress. Evidence-based behavior guidance techniques such as positive reinforcement, tell-show-do, and verbal reassurance rely heavily on verbal communication and may be less effective when language barriers exist [7]. Furthermore, the clinician’s own difficulty in understanding a patient with a different primary language may lead to a biased or lower classification of the child’s behavior, potentially even subconsciously. These results underscore the vital role of language in a clinician’s ability to positively influence a child’s behavior, enabling them to maintain cooperation even when experiencing dental anxiety. Dental anxiety and dental behavior management problems are two different concepts related to each other but not identical [5]. In children, dental behavior management problems were found to be significantly associated with high dental anxiety in both retrospective and prospective studies [24].

The findings of the present study suggest that language concordance does not have a differential impact on post-examination anxiety across the age range studied or by gender. In the existing literature, the relationship between gender, dental anxiety, and behavioral problems remains inconclusive. While some studies have identified a correlation between female gender and higher anxiety levels [5,25], the results of the current study do not support such an association.

Beyond the linguistic challenges, significant cultural differences may have also influenced the study’s outcomes. Language disparities often encompass a spectrum of values, beliefs, and practices. The educational values and social perspectives inherent to different sectors can sometimes foster a sense of apprehension or aversion toward perceived others [26,27]. Consequently, a pediatric patient’s first encounter with an individual from a different cultural background might occur in the dental office, potentially elevating their initial threshold of anxiety. In fact, it is plausible that many children who were potential candidates for this study were excluded during the initial screening for this very reason (i.e., presenting with high baseline anxiety due to the cultural novelty of the encounter).

The present study has several limitations that should be acknowledged. First, the relatively small sample size (N = 60) and the fact that the study was conducted at a single medical center may limit the generalizability of the findings. A larger, multicenter study might yield different results, potentially affecting the statistical significance and the broader applicability of the data. Second, the study was restricted to patients with low-to-moderate baseline anxiety (FIS scores of 1–3). This inclusion criterion may have introduced a selection bias, meaning that the findings cannot be generalized to children with high dental anxiety. The responses of highly anxious children to language barriers might differ significantly, as their pre-existing fear may overshadow or interact differently with communication challenges. Future research should consider including patients with higher baseline anxiety levels to explore these dynamics more comprehensively. Third, since the dentists who rated the children’s behavior were not blinded to group allocation, there is a potential risk of observer bias. Complete blinding was not feasible due to the nature of the intervention, as the clinicians had to actively converse with the patients to conduct the examination. To mitigate this bias, standardized and well-defined scoring criteria were strictly applied. Nonetheless, future studies might consider utilizing independent, blinded video evaluators to assess patient behavior retrospectively. Fourth, within the study group, interpretation was primarily provided by parents. This prevented meaningful analysis of the no-interpretation scenario and comparison between different types of translators. Also, parental dental anxiety was not measured, which represents a potential unmeasured confounding factor that could have influenced children’s behavioral responses and anxiety. Subsequent studies should investigate the impact of different types of interpreters, such as professional medical interpreters or dental staff, as well as scenarios where no interpretation is available. Fifth, this research focused exclusively on the dental examination. Given that operative dental treatment (e.g., restorative or surgical procedures) is typically more invasive and anxiety-inducing, future studies should evaluate the influence of language discordance on child anxiety and behavior during dental treatment. The effectiveness of specific behavior guidance techniques (such as tell-show-do, positive reinforcement, and modeling) in the context of language barriers should also be investigated. Sixth, the clinical examinations were conducted by ten different pediatric dentistry residents, and formal inter-examiner calibration was not performed. This may introduce variability regarding individual non-verbal communication skills and behavioral scoring. However, all examiners were senior residents trained uniformly within the same department with extensive daily experience utilizing the Frankl scale. While this multi-provider setup represents a potential limitation, it simultaneously reflects real-world clinical practice in institutional settings, thereby enhancing the external validity of the findings. Finally, non-verbal communication strategies were not formally documented or measured in this study, despite their likely importance in bridging the linguistic gap. Future research should systematically assess the use and effectiveness of non-verbal communication techniques in language-discordant pediatric dental encounters.

## 5. Conclusions

The primary findings of this pilot study indicate that, within the context of non-invasive clinical and radiographic dental examinations, language discordance between the dentist and the pediatric patient does not significantly increase anxiety levels or negative behavior in this sample. These results suggest that non-verbal communication and the presence of a bilingual parent serving as an interpreter are likely mediating factors in mitigating anxiety within the pediatric population when language barriers exist. However, these findings are preliminary and non-generalizable and must be interpreted with caution, as potentially confounding variables, such as the children’s prior dental experiences, parental dental anxiety, and specific cultural backgrounds, were not fully controlled. To address these deficiencies and limitations, further well-controlled studies involving larger, anxious pediatric populations should employ a prospective design featuring rigorously calibrated, blinded examiners, comprehensive pre-treatment behavioral and anxiety screening for both parents and children, and a balanced enrollment strategy to systematically evaluate the distinct clinical trajectories of professional interpretation, ad hoc interpretation, and language-discordant care without translation during more invasive restorative dental treatment.

## Figures and Tables

**Table 1:** Study data for the control and study groups (N = 60).

Variable	Study Group		Control Group		*p*-Value
	(*n* = 30)		(*n* = 30)		
	Mean	SD ^1^	Mean	SD ^1^	
Age (years)	6.17	1.91	6.97	1.92	0.11
Baseline anxiety (FIS)	2.03	0.87	1.87	0.9	0.478
Post-examination anxiety (FIS)	1.82	1.04	1.9	1.09	0.99
Anxiety change	−0.21	1.11	0.03	1.27	0.646
Behavior during clinical examination (Frankl)	3.66	0.48	3.53	0.68	0.871
Behavior during radiographic examination (Frankl)	3.62	0.65	3.71	0.69	0.615

^1^ SD = standard deviation.

## Data Availability

The raw data supporting the conclusions of this article will be made available by the authors on request.

## Supplementary Materials

The following supporting information can be downloaded at: https://www.mdpi.com/article/10.3390/dj14070448/s1, Table S1: Raw research dataset of patient demographics, clinical characteristics and anxiety scores.

## Abbreviations

The following abbreviations are used in this manuscript:

EU	European Union
FIS	Facial Image Scale
SD	Standard deviations
